# Supplementary material for: Current Societal Views about Sustainable Wildlife Management and Conservation: A Survey of College Students in China
Source: Animals (Basel). 2020 Oct 6;10(10):1821. doi: 10.3390/ani10101821 (PMC7599632; doi:10.3390/ani10101821)
Supplement: Supplementary file 1 [file animals-10-01821-s001.pdf]

animals

Supplementary Materials: Current Societal Views about Sustainable Wildlife Management and Conservation: A Survey of College Students in China

Zhen Miao [1], Qiang Wang [2], Dongxiao Chen [1], Zhifan Song [1], Wei Zhang [1,*], Xuehong Zhou [1,*] and Douglas C. MacMillan [3]

1 College of Wildlife and Protected Area, Northeast Forestry University, Harbin 150040, China; miaozhen43566@163.com (Z.M.); dongxiao_c@163.com (D.C.); 13136751029@163.com (Z.S.)
2 Key Laboratory of Wetland Ecology and Environment, Northeast Institute of Geography and Agroecology, Chinese Academy of Sciences, Changchun 130102, China; qwang@neigae.ac.cn
3 Durrell Institute of Conservation and Ecology (DICE), University of Kent, Canterbury, Kent CT2 7NR, UK; dcm@kent.ac.uk
* Correspondence: zwfur@nefu.edu.cn (W.Z.); xuehong_zhou2012@nefu.edu.cn (X.Z.)

Received: 12 August 2020; Accepted: 02 October 2020; Published: date

Table S1. Demographics characteristics of participants.

Demographics	Abbreviation	Category	Percentage (%)
Gender	Gender	Male	50.9
		Female	49.1
Grade	Grade	Freshman	47.6
		Sophomore	20.0
		Junior	17.5
		Senior	6.60
		Postgraduate	8.30
Major	Major	Agriculture	3.60
		Science	28.3
		Engineering	24.0
		Medicine	8.10
		Economics	6.60
		Management	8.10
		Law (arts)	6.30
		Literature (arts)	14.9
		Fine art (arts)	0.20
Whether Vegetarian or not?	Vegetarian	Yes	22.6
		No	77.4
	Attention	Yes	60.8

Have you paid attention to information related to wildlife conservation?		No	39.2
Have you taken environmental protection electives during university?	Electives	Yes	34.4
		No	65.6
Have you participated in activities about wildlife?	Activity	Yes	28.2
		No	71.8
Ways to learn about wildlife	Ways	TV	54.2
		Broadcast	16.6
		Internet	60.1
		Newspaper	33.9
		School	30.9
		Family	10.8
		Friends	14.2
		Others	12.0

Table S2. Summary of the classification and regression tree (CART) models of seven categories of issues.

Question	Factor	Result
Release	Primary: vegetarian<br>Secondary: electives, activity<br>Tertiary: grade	Vegetarian was the most important predictor for the Release Problem, with vegetarian students (0.769) scored lower than non-vegetarian students (1.253). Among non-vegetarian students, at the second node those who had taken electives about environmental conservation (1.092) had lower score than other students (1.322); for vegetarian students, those who had participated in activities about wildlife (0.505) scored lower than students who had not (0.986). For non-vegetarian students who had taken electives, at the third node freshmen (0.619) scored lower than students from higher grade (1.277). Overall, non-vegetarian students who had participated in activities about wildlife had the lowest score (0.505).
Animal Welfare and Rights	Primary: attention<br>Secondary: major	At the first node, attention was the most important predictor for the Animal Welfare; students who had paid attention to wildlife conservation (-0.345) scored lower than students who had not (-0.173). Among students who had not paid attention to wildlife conservation, at the second node students majoring in agriculture, art, law (-0.141) scored higher than engineering, science, economics, management, medicine, fine art (-0.418).
Utilization and Wildlife Conservation	Primary: vegetarian<br>Secondary: major	Vegetarian was the most important predictor for the Utilization and Wildlife Conservation, with non-vegetarian students (0.281) scored higher than vegetarian students (-0.101). Among vegetarian students, at the second node students majoring in science, engineering, agriculture, fine art (0.191) scored higher than students majoring in economics, art, law, management and medicine (-0.432).
Wildlife Management	Primary: grade<br>Secondary: gender	Grade was the most important predictor for the Wildlife Management; freshmen and graduate students (0.679) scored lower than sophomores, juniors and seniors (1.165). Among sophomores, juniors and seniors, at the second node females (1.005) had lower score than males (1.301).
Vegetarianism and Wildlife Conservation	Primary: vegetarian<br>Secondary: activity	Vegetarian was the most important factor for the Vegetarianism and Wildlife Conservation, with non-vegetarian students (0.714) had higher score than vegetarian students (0.265). Among non-vegetarian students, at the second node those who had participated in activities about wildlife scored lower (0.431) than students who had not (0.799). Overall, non-vegetarian students without participating in activities had the highest score (0.799).
Public and Wildlife Conservation	Primary: elective<br>Secondary: gender, grade	Elective was the most important predictor for the Public and Wildlife Conservation; students who had taken environmental electives (0.892) had lower score than students who had not (1.204).At the second node, the predictors become more diverse, including

		gender and grade; among students without taking electives, females (1.311) scored higher than males (1.090); and for students who had taken electives, sophomores and juniors (1.089) scored higher than seniors, freshmen and graduate students (0.708).
Trophy Hunting	Primary: grade<br>Secondary: attention	Grade was the most important predictor for the Hunting Issues, with freshmen and graduate students (-0.405) scored lower than students from other grades (-0.035). Among freshmen and graduate students, at the second node those who had paid attention to wildlife conservation (-0.512) scored lower than students who had not (-0.230).

The classification and regression tree model on the seven categories of issues

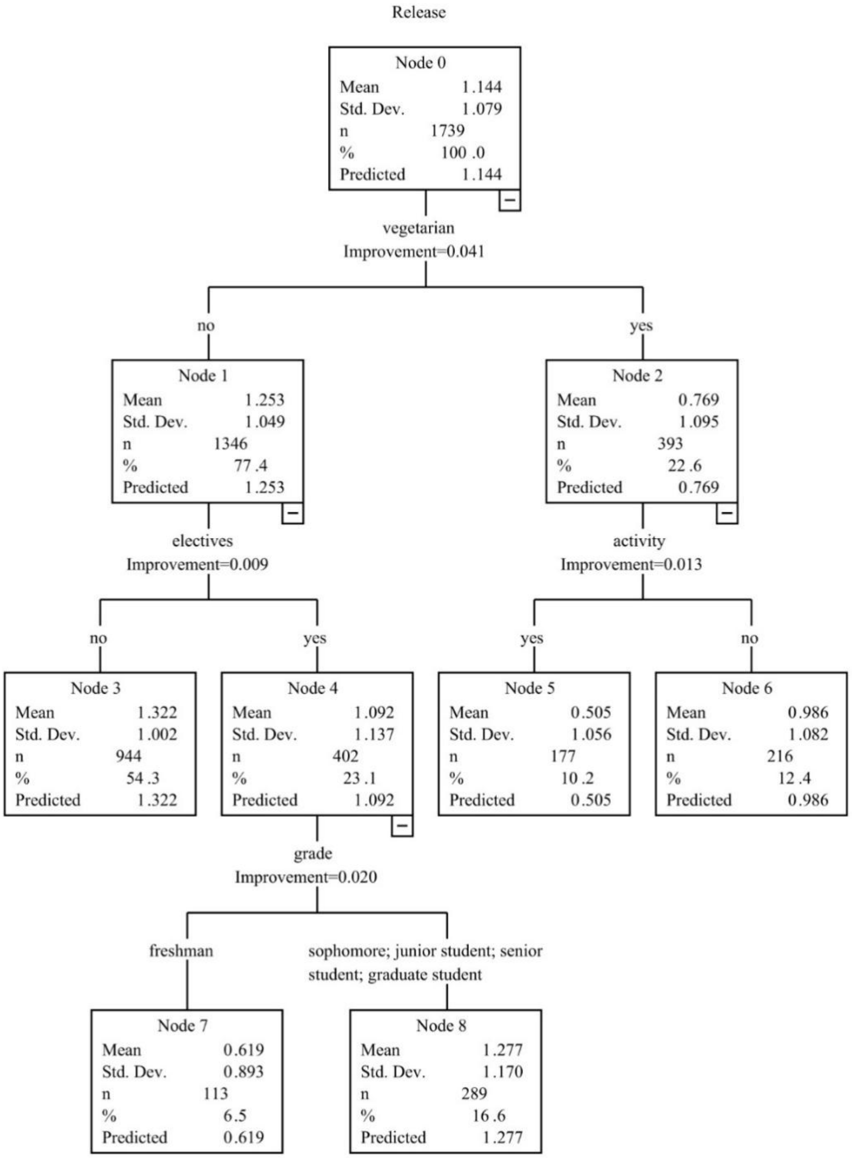

Figure S1. The classification and regression tree model for the Release.

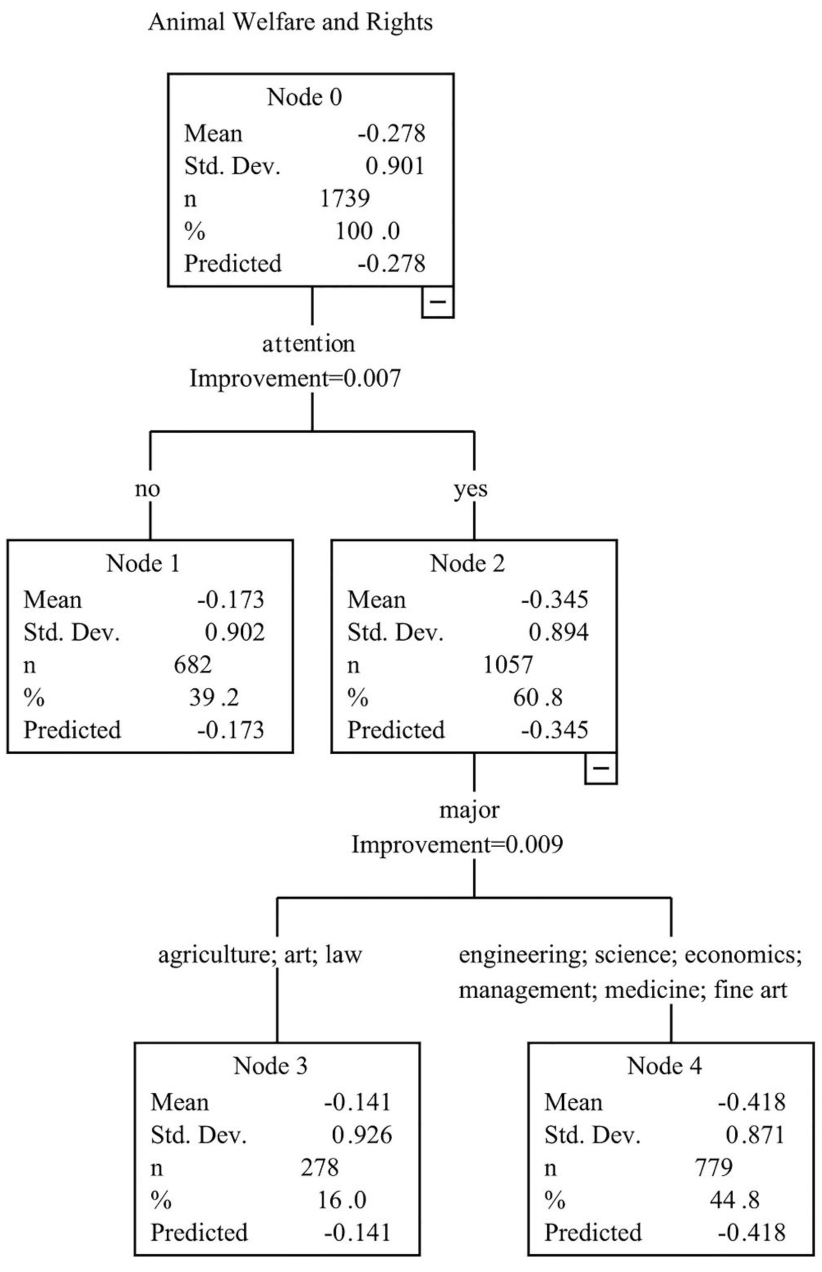

Figure S2. The classification and regression tree model for the Animal Welfare and Rights.

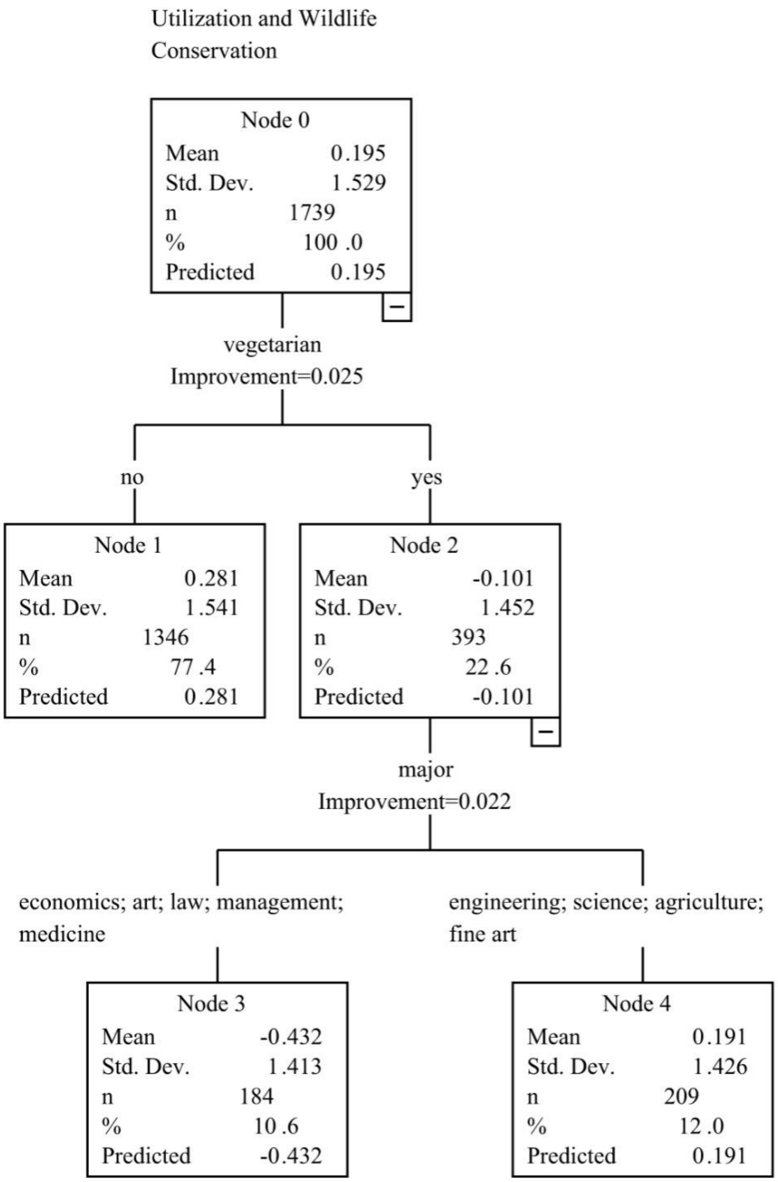

Figure S3. The classification and regression tree model for the Utilization and Wildlife Conservation.

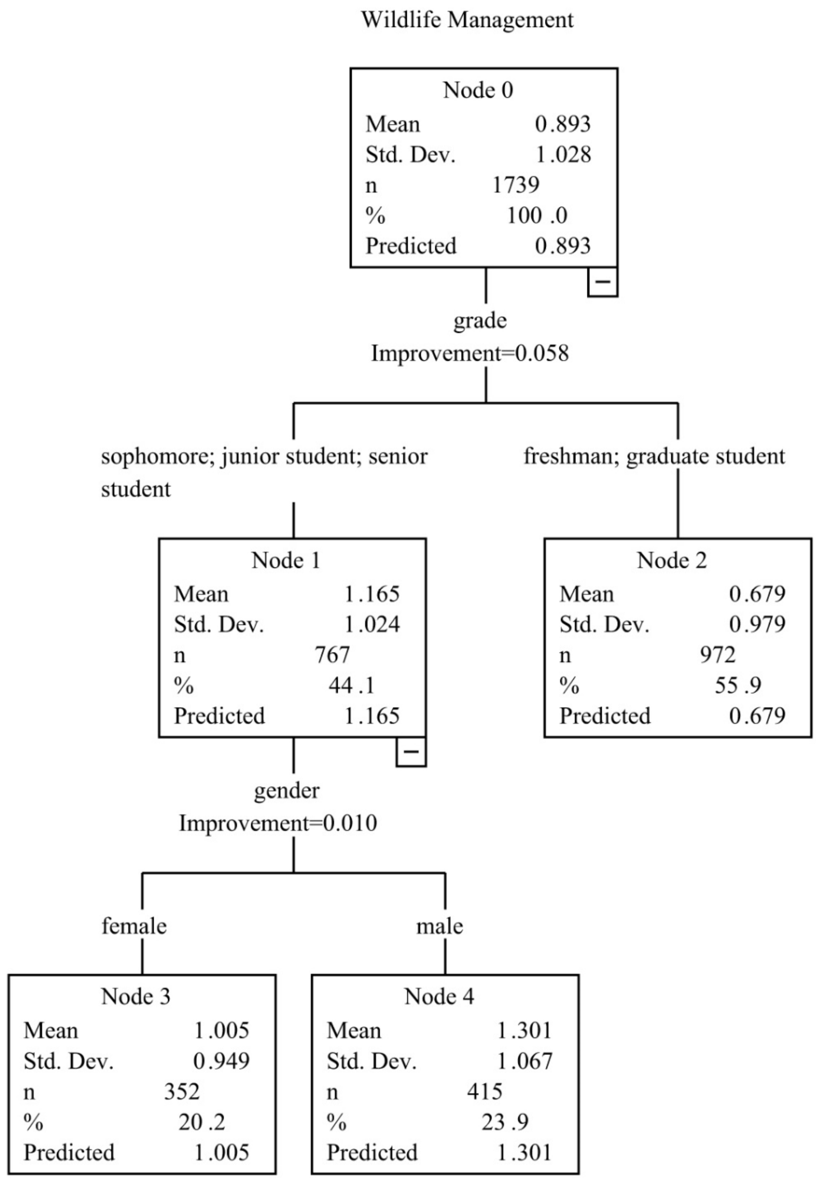

Figure S4. The classification and regression tree model for the Wildlife Management.

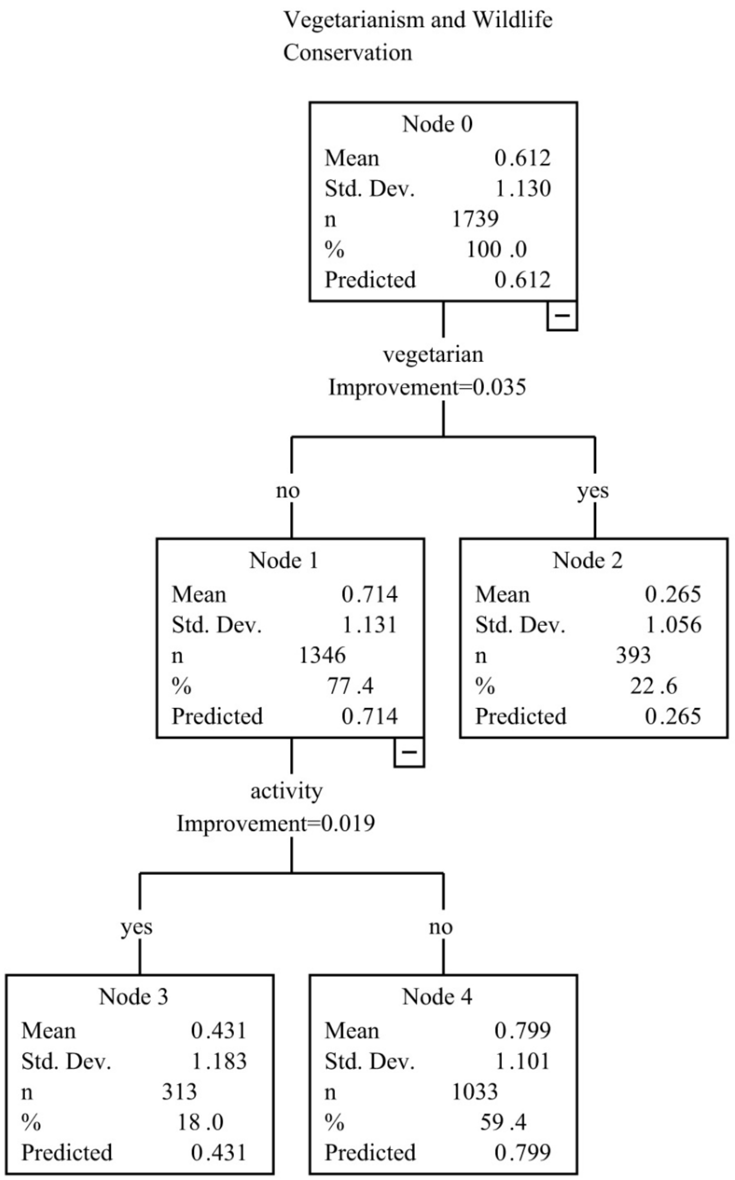

Figure S5. The classification and regression tree model for the Vegetarianism and Wildlife Conservation.

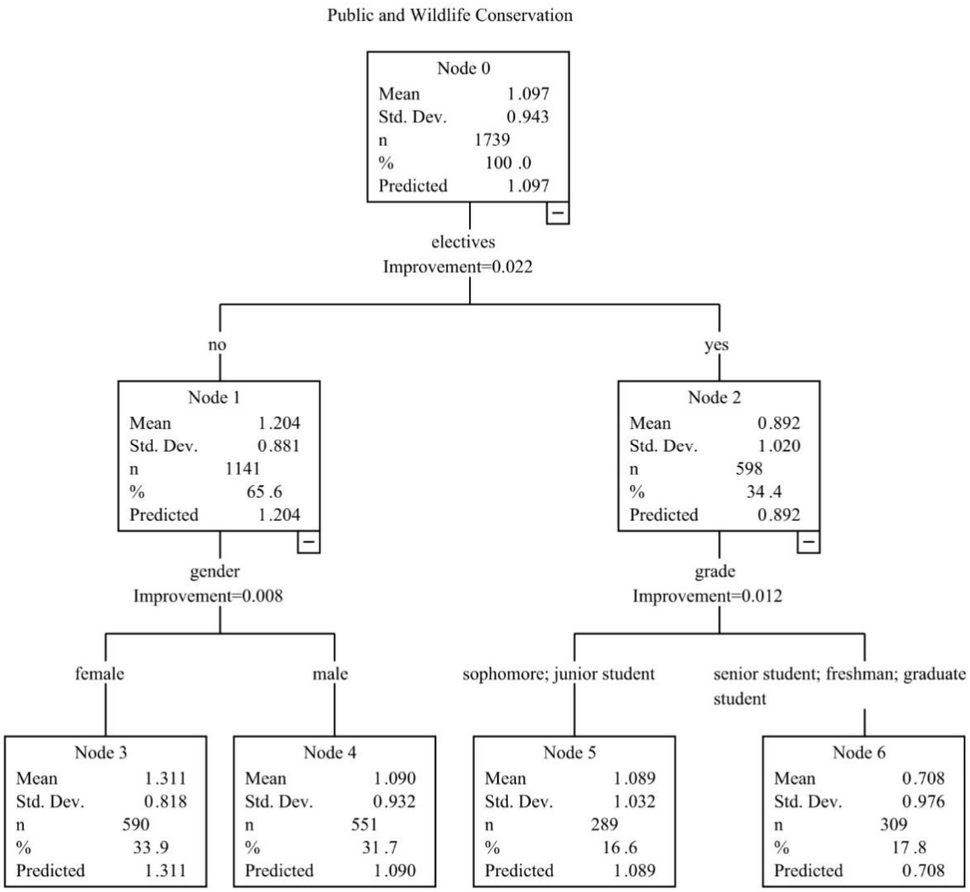

Figure S6. The classification and regression tree model for the Public and Wildlife Conservation.

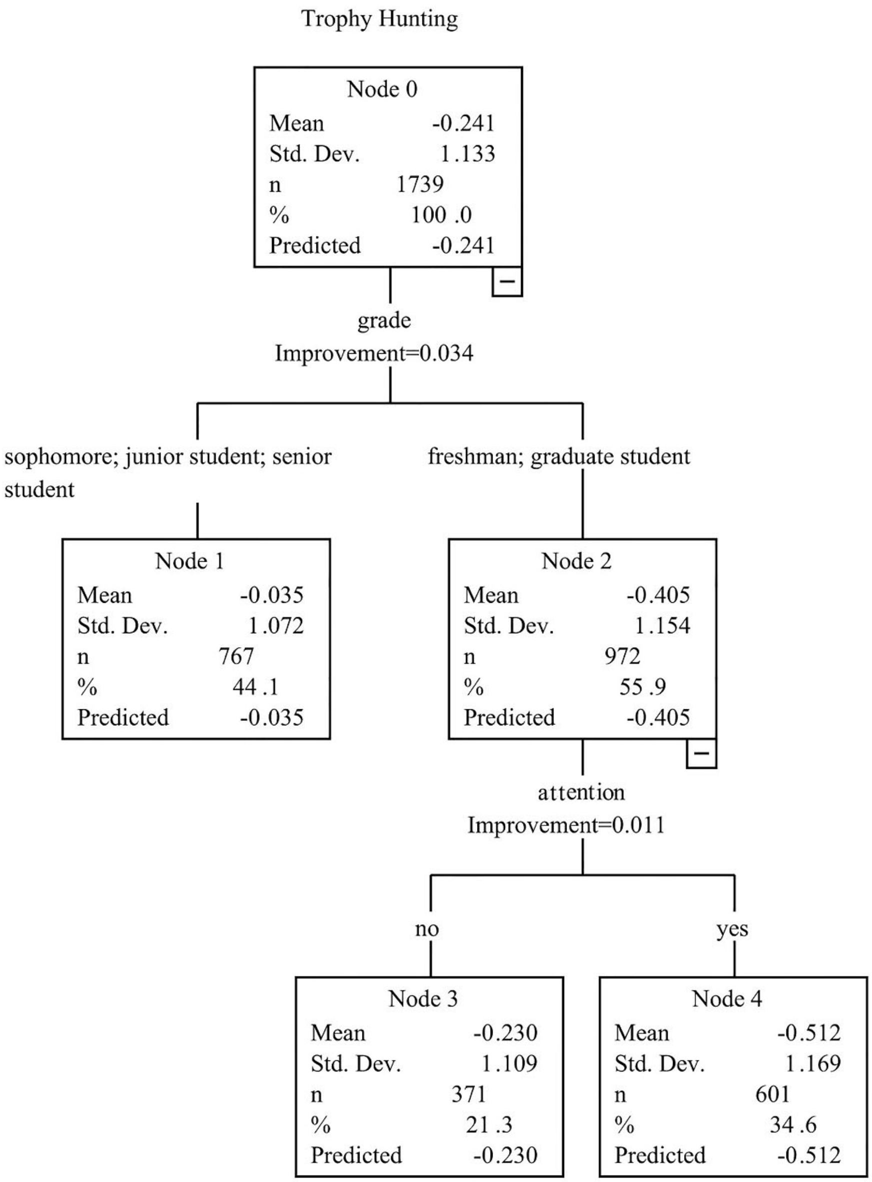

Figure S7. The classification and regression tree model for the Trophy Hunting.
